# The interaction of hepatitis B virus with the ubiquitin proteasome system in viral replication and associated pathogenesis

**DOI:** 10.1186/s12985-019-1183-z

**Published:** 2019-05-30

**Authors:** Fanyun Kong, Hongjuan You, Delong Kong, Kuiyang Zheng, Renxian Tang

**Affiliations:** 1 Jiangsu Key Laboratory of Immunity and Metabolism, Department of Pathogenic Biology and Immunology, Xuzhou Medical University, Xuzhou, 221004 Jiangsu China; 2 National Demonstration Center for Experimental Basic Medical Sciences Education, Xuzhou Medical University, Xuzhou, 221004 Jiangsu China

**Keywords:** Hepatitis B virus, Ubiquitin, Proteasome, Pathogenesis, Replication

## Abstract

**Background:**

The ubiquitin proteasome system (UPS) regulates the expression levels of cellular proteins by ubiquitination of protein substrates followed by their degradation via the proteasome. As a highly conserved cellular degradation mechanism, the UPS affects a variety of biological processes and participates in viral propagation.

**Main body:**

During hepatitis B virus (HBV) infection, the UPS is shown to act as a double-edged sword in viral pathogenesis. On the one hand, the UPS acts as a host defense mechanism to selectively recognize HBV proteins as well as special cellular proteins that favor the viral life cycle and induces their ubiquitin-dependent proteasomal degradation to limit HBV infection. On the other hand, the HBV has evolved to subvert the UPS function for its own advantage. Moreover, in the infected hepatocytes, certain cellular proteins that are dependent on the UPS are involved in abnormal biological processes which are mediated by HBV.

**Conclusion:**

The molecular interaction of HBV with the UPS to modulate viral propagation and pathogenesis is summarized in the review. Considering the important role of the UPS in HBV infection, a better understanding of the HBV-UPS interaction could provide novel insight into the mechanisms that are involved in viral replication and pathogenesis and help to develop potential treatment strategies targeting the UPS.

## Background

As a highly transmissible pathogen, the hepatitis B virus (HBV) is a major public health threat and causes variable degrees of liver diseases, including acute and chronic hepatitis, liver fibrosis, cirrhosis, and hepatocellular carcinoma (HCC) worldwide [1]. The genome of the HBV is a 3.2 kb partially double-stranded DNA, which has 4 overlapping open reading frames (ORFs) termed as S, C, P, and X. The S ORF has preS1, preS2 and S coding regions that encode 3 viral envelope proteins, including LS (large surface), MS (medium surface), and S (small surface) proteins; the C ORF contains C and precore genes that are responsible for the expression of viral core protein (HBc), and precore protein, which could be cleaved in its C-terminal part and then secreted as HBe antigen; P and X ORFs encode viral polymerase (Pol protein) and non-structural protein HBx [2, 3]. After entry into hepatocytes via the sodium taurocholate cotransporting polypeptide (NTCP) [4], the HBV genome is delivered to the nucleus and converted into a covalently closed circular DNA (cccDNA). Sequentially, the cccDNA forms a minichromosome and serves as the template for transcription of distinct viral transcripts, including 3.5 kb preC mRNA and pregenomic RNA (pgRNA), two envelope mRNAs (2.4 and 2.1 kb), and X mRNA (0.7 kb) [5–7]. Among the viral transcripts, preC mRNA encodes precore protein. pgRNA translates viral HBc and Pol proteins, and also acts as a template for the replication of the HBV genome. The 2.4 and 2.1 kb envelope mRNAs encode LS, MS, and S proteins. In addition, the 0.7 kb X mRNA translates HBx protein [8]. After translation of viral RNAs into HBV proteins occurs in the host cytoplasm, viral pgRNA is encapsulated into core particles. Inside the core particle, pgRNA is further reversely transcribed into viral DNA. Then, mature viral particles containing HBV DNA are enveloped and released from host cells [9].

Since the HBV is a small DNA virus, and there is only limited genetic information in the viral genome, the virus heavily relies on cellular factors for viral replication. During HBV infection, a variety of cellular factors are recruited by the virus to regulate multiple steps in the HBV replication cycle [10]. Moreover, the HBV is capable of selectively and specifically altering the expression of intracellular factors, which are involved in the host immune response, to mediate persistent viral infection [11]. More importantly, the cellular factors affected by the HBV also modulate various biological processes, including innate immune response, cell cycle, proliferation, apoptosis, and invasion, and play vital roles in the development of liver diseases [12].

Until now, the molecular mechanisms related to HBV replication and associated liver diseases have not been well understood. Currently, cumulative evidence indicates that the host ubiquitin proteasome system (UPS) has vital roles in HBV replication as well as virus-related pathogenesis. As one type of post-translational modification (PTM), the main function of the UPS is mediating the degradation of cellular proteins. In general, the proteins are first labeled with a small but highly conserved protein ubiquitin (Ub) (a process named ubiquitination) and then the labeled proteins are further recognized and degraded by the proteasome. During ubiquitination, one or more Ub molecules are covalently attached to target proteins via a series of cascade reactions catalyzed by different enzymes, including Ub-activating enzyme E1, Ub-conjugating enzyme E2, and Ub ligase (E3). At first, in an ATP-dependent manner, Ub is activated and a high-energy thioester bond is formed with E1; next, Ub is transferred from E1 to E2 with the formation of a thioester bond between Ub and E2. Sequentially, E3 transfers Ub from E2 to the lysine residue of protein substrates. In humans, two E1 proteins, around forty E2 proteins, and several hundred E3 ligases exist and regulate Ub-mediated proteolysis [13]. After multiple rounds of ubiquitination, a poly-Ub chain is formed on the target protein substrate, and then the recognition and degradation of the target protein substrate is accomplished by the proteasome. Like other types of PTM, ubiquitination is reversible. Ub could be removed from the target protein substrate with the help of deubiquitinating enzymes (DUBs). In addition, the human genome encodes about 86 Ub-specific DUBs [14], which have the function of altering the level of ubiquitination in protein substrates [15–17].

It has become increasingly evident that the UPS could selectively recognize viral proteins and induce their ubiquitination and degradation to control virus infection [15]. Conversely, different viruses have developed sophisticated mechanisms to subvert the UPS function for their own benefit [17]. In addition, the cellular proteins degraded by the UPS have a variety of biological roles [15, 16], and some of them regulated by the viruses using the UPS are responsible for the abnormal function of infected cells. Here, we summarize the available data about the molecular interaction of the HBV with host UPS to regulate the expression of HBV proteins and special cellular proteins that modulate viral replication and pathogenesis.

## Main text

### The role of the UPS in viral protein regulation of HBV

**Fig. 1:**
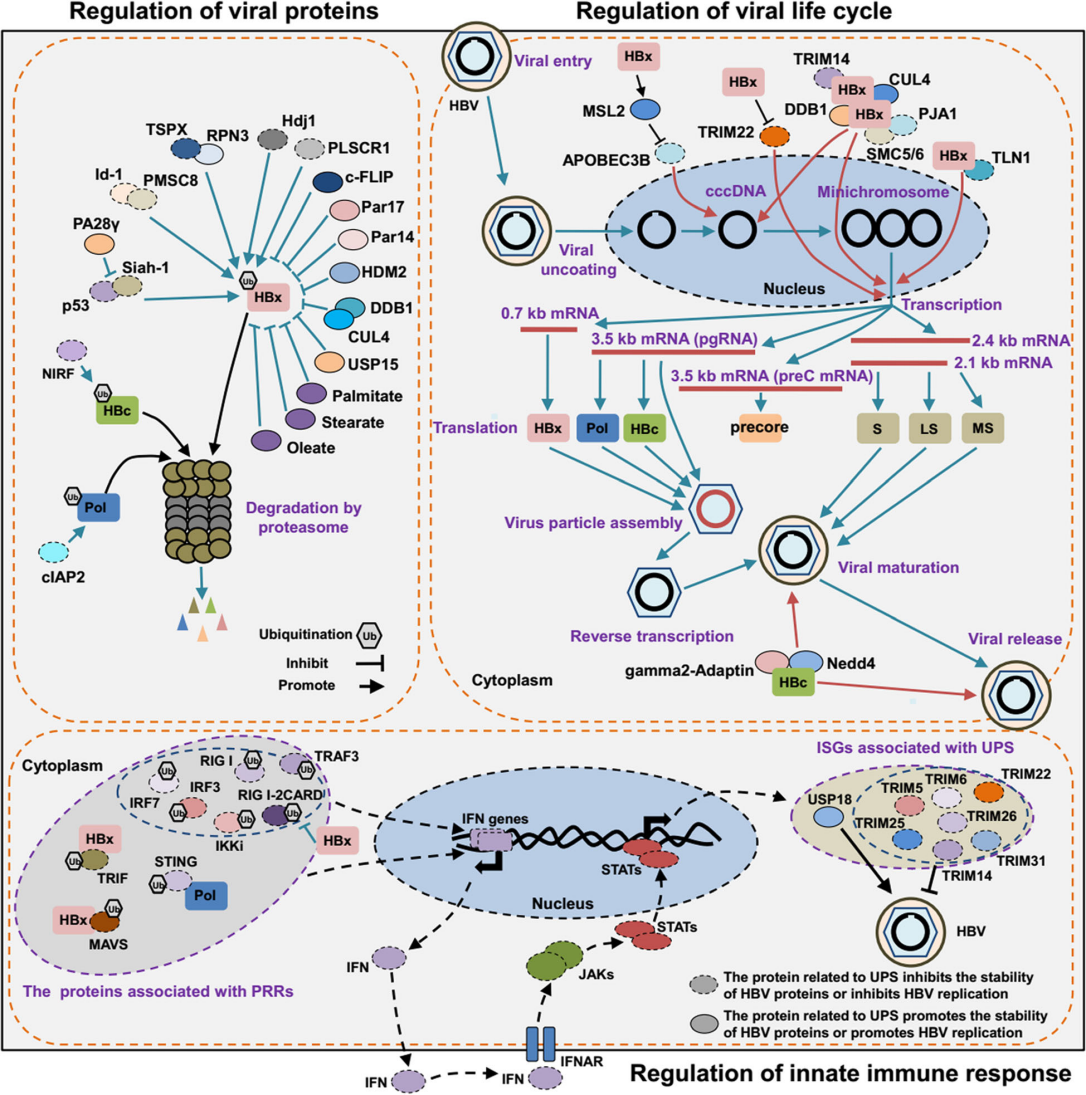
The mechanisms associated with the UPS in HBV replication. The UPS modulates the replication of the HBV via the regulation of viral proteins, the viral life cycle, and innate immune response. Several components of the UPS, such as P53 and Id-1, could repress viral replication through proteasome-dependent degradation of HBV proteins, including HBx, HBc, and Pol. However, some specific proteins interact with HBV proteins to inhibit the degradation of viral proteins. The UPS participates in different steps of the viral life cycle, including the stability of cccDNA, transcription and maturation, by interacting with HBx or HBc. Many components of the UPS participate in the innate immune response to inhibit viral replication. HBV proteins, such as HBx and Pol, promote the replication of the virus via regulating the ubiquitination of proteins associated with the innate immune response. PRRs: pattern recognition receptors. ISGs: IFN stimulated genes

It is well-known that virus-encoded proteins are essential for viral replication. However, multiple studies have shown that the UPS serves as an anti-viral mechanism through selective degradation of viral proteins [18–23]. Until now, different HBV proteins have been reported to be degraded by the UPS (Fig. 1). For example, Np95/ICBP90-like RING finger protein (NIRF), an E3 ligase, could bind to HBc and promote its ubiquitination and degradation to decrease the levels of HBc protein [18]. Cellular inhibitor of apoptosis protein 2 (cIAP2), which has a carboxy-terminal RING finger domain with the activity of E3 ligase, is able to promote the degradation of HBV Pol protein mediated by the UPS [20].

Different laboratories have investigated the role of HBx on viral replication. Owing to the lack of a natural model of HBV infection previously, the role of HBx in the complete virus life cycle has not been well-established. Given this limitation, most reported studies, including the studies using a plasmid-based HBV replication system and associated animal models, strongly support the importance of HBx for virus replication [24]. Currently, HepG2 cell overexpressing the HBV entry receptor NCTP (HepG2-NTCP) has been established, and was highly susceptible to wide-type HBV infection [25, 26]. In addition, it was found that the virus with a defective mutation of HBx fails to produce the detectable levels of HBV replication after infecting HepG2-NTCP cells [27]. Using the cell model, Ko et al. also investigated the infection kinetics of HBV during long-term culture, and the research showed that the kinetics of intracellular HBx expression was followed the viral cccDNA dynamics and reaches maximum levels earlier than other viral proteins [28]. Together, these preliminary results imply that HBx is essential for the natural course of HBV infection, and could serve as an early viral protein that is needed immediately for viral replication. In general, HBx is found to be produced at a very low level in chronic hepatitis patients with HBV infection, because it was found to be rapidly degraded by the UPS pathway in the host cells [29, 30]. Furthermore, recent studies have indicated that several special cellular proteins as restriction factors limit HBV replication by repressing the expression of HBx via the UPS. For example, the tumor suppressor p53 could induce Ub-dependent proteasomal degradation of HBx [31], through an E3 ligase, seven in absentia homologue 1 (Siah-1) [22]. Ling et al. showed that Id-1 [32], a member of the HLH protein family, was capable of interacting with proteasome subunit C8 (PMSC8), to facilitate the degradation of HBx. In addition, the X-linked tumor suppressor TSPX was found to bind with the proteasome component RPN3 and reduce the stability of HBx via the proteasome-dependent degradation [33]. It was also shown that Hdj1 and phospholipid scramblase 1 (PLSCR1) could promote HBx degradation by ubiquitination and a proteasome-dependent mechanism to repress viral replication [34, 35]. More importantly, six lysines in HBx protein were found to be ubiquitinated [23]. However, how these factors as mentioned above selectly target these ubiquitination sites of HBx is largely unknown. Therefore, further experiments are warranted to elucidate the details related to HBx ubiquitination mediated by different cellular factors.

Host cells could utilize the UPS to degrade viral proteins and restrict viral growth, but the HBV can manipulate many specific proteins to limit the degradation of viral proteins mediated by the UPS pathways. For example, although the turnover of HBx is rapid, the reported studies indicated that the bimodal half-life of HBx was influenced by the intracellular location of the protein [24], and it was speculated that the interaction of HBx with other intracellular proteins in a different cell location may be necessary for the stability of the HBx protein. In addition, several proteins have been reported to control HBV replication via the interaction with HBx to regulate the stability of the protein through UPS-associated mechanisms. For example, damaged DNA binding protein 1 (DDB1) and E3 ligase CUL4 could interact with HBx, forming a HBx-DDB1-CUL4 E3 ligase complex, and protect the viral protein from proteasome-mediated degradation to promote HBV replication [36–38]. Additionally, Liu et al. showed that the E3 ligase HDM2 (also known as MDM2) promoted the NEDDylation of HBx to enhance HBx stability by blocking its degradation mediated by ubiquitination [39]. The study from Yeom et al. indicated that proteasomal activator 28 gamma (PA28γ) could down-regulate the expression of Siah-1 and p53 to facilitate the inhibition of HBx degradation [40]. Besides, it is found that cellular FLICE inhibitory protein (c-FLIP) could interact with HBx and protect it from Ub-dependent degradation to maintain HBx stability [41]. Saeed et al. showed that parvulin 14 (Par14) and parvulin 17 (Par17) proteins enhanced the stability of HBx via the direct interactions with the viral protein [42]. Fatty acids, including palmitate, oleate, and stearate, were also found to increase the stability of HBx protein by preventing its proteasome-dependent degradation [43]. Su et al. found that deubiquitylating enzyme Ub-specific protease 15 (USP15) directly interacted with HBx to reduce the ubiquitination and proteasomal degradation of the viral proteins [44]. Taken together, these evidence indicate that the UPS could be used to control the degradation or stability of HBc, Pol, and HBx protein. However, the molecular mechanisms associated with expression of viral envelope proteins regulated by the UPS remain unknown and require additional studies to elucidate.

### The role of the UPS in the viral life cycle to regulate HBV replication

The HBV life cycle involves cell entry, viral genome uncoating, replication, transcription, protein expression, reverse transcription, viral particle maturation, and release (Fig. 1). So far, an abundance of studies, using different experimental systems including the cultured cells transfected with plasmid containing a greater-than-unit-length HBV genome, have indicated that several components of the UPS, such as PJA1 [45] and SMC5/6 [46], could serve as restriction factors to inhibit the replication cycle of the HBV.

However, during HBV infection, virus-encoded proteins have evolved to interact with host UPS, and could regulate the expression or stability of cellular molecules associated with host UPS to meet the requirement for virus replication in various steps of the life cycle (Fig. 1). Based on hepatoma cells or primary hepatocytes transiently transfected with a plasmid encoding the HBV genome expressing HBx, or the plasmid that contains the mutations preventing HBx expression [47], HBx was found to play vital roles in different steps of HBV replication. Furthermore, HBx could affect viral replication via a UPS-dependent pathway [48]. For example, Gao et al. found that HBx could activate and enhance the stability of HBV cccDNA via increasing the expression of MSL2, an E3 ligase that has the capability of reducing the protein level of APOBEC3B [49]. SMC5/6 has been reported to have the capability of inhibiting HBV transcription [46]. Furthermore, PJA1, an E3 ligase, binds to SMC5/6 and facilitates the protein complex to eliminate cccDNA to inhibit HBV replication [45]. As mentioned above, HBx could interact with DDB1 and CUL4 to form a HBx-DDB1-CUL4 E3 ligase complex, and based on the interaction with DDB1 and CUL4, HBx could stimulate viral transcription [37]. Using substrate-trapping proteomics, Murphy et al. identified that SMC5/6 were the substrates of the HBx-DDB1-CUL4 E3 ligase complex, and HBx degraded SMC5/6 through ubiquitination and the proteasomal pathway to enhance HBV replication [27]. In addition, the study from Klundert et al. reported Talin-1 (TLN1) was a viral restriction factor that could repress the transcription of the HBV, but HBx was able to relieve its restriction by inducing the degradation of TLN1 mediated by the proteasome pathway to facilitate HBV replication [50]. In addition, multiple TRIM proteins, which have the function of E3 ligase, including TRIM5, TRIM6, TRIM11, TRIM14, TRIM22, TRIM25, TRIM26, TRIM31, and TRIM41, have been shown to repress HBV transcription [51, 52]. However, HBx could decrease the expression of TRIM22 at the gene level to facilitate the transcription of viral genes [53]. In summary, these mentioned studies demonstrate that HBx could promote the replication of HBV via regulating the expression and function of the components of host UPS.

Apart from HBx, HBc has been shown to play a critical role in viral maturation and release [54], but the precise mechanisms have not been well-assessed. Rost et al. found that HBc could interact with gamma2-Adaptin, an Ub-interacting adaptor, and Nedd4, an E3 ligase, to enhance assembly and particle release of the virus [55]. However, whether the components of the UPS participate in other steps of the viral life cycle, such as genome uncoating during HBV infection, is still unclear.

### The role of the UPS in the innate immune response to regulate HBV replication

The host innate immune response is the first line of defense against viral infection. Via recognizing viral pathogen-associated molecular patterns (PAMPs) by pattern recognition receptors (PRRs), including NOD like receptors (NLRs), toll-like receptors (TLRs), nucleic acid sensing PRR includes the stimulator of interferon genes (STING) and RIG-I like receptors (RLRs), the host cells trigger special signals to activate intracellular pathways and induce the production of interferon (IFN) and cytokines to clear the virus [11, 56]. However, during infection with HBV, the virus does not trigger or only triggers a very limited innate immune response to cause persistent infection [57]. The exact mechanisms of the inhibition of the innate immune response mediated by the HBV are not well-understood. Until now, several studies from different laboratories found that the HBV had evolved to use the UPS to interfere with the expression of PRRs-associated proteins (Fig. 1), to act against the antiviral immune response. For example, Khan et al. demonstrated that the HBV was able to induce Parkin, an E3 ligase, which had the capability of recruiting the linear Ub assembly complex (LUBAC) to mitochondria, bonding to mitochondrial antiviral signaling (MAVS), and accumulating unanchored linear polyubiquitin chains on MAVS protein through LUBAC, to block the synthesis of IFN-β [58]. In addition to these, current researches demonstrate that, among the HBV proteins, HBx could utilize the UPS to inhibit the innate immune response induced by the virus. For example, Jiang et al. indicated that HBx could inhibit the ubiquitination of multiple proteins that associate with PRRs, such as IRF3, IRF7, RIG I, RIG I-2CARD, TRAF3, and IKKi, to inhibit the production of IFN and facilitate viral replication [59]. In addition, in HBV-infected cells, HBx has been shown to interact with MAVS and promote the degradation of MAVS mediated by the proteasome, to prevent the production of IFN-β [60]. HBx also reduces the expression of TIR-domain-containing adaptor inducing interferon-beta (TRIF) protein via the proteasomal pathway to inhibit the activation of TLR signals [61]. Apart from HBx, HBV Pol protein was also found to interact with STING, and disrupt the ubiquitination of STING to inhibit the activation of STING-stimulated IRF3 as well as the induction of IFN-β [62].

IFN, an immune molecule that could be induced by PRRs, has been successfully used for clinical treatment of patients with HBV infection. However, the molecular mechanisms of the anti-HBV effect mediated by IFN are not completely understood. It is well-accepted that, upon binding to the cellular receptors IFNAR, IFN could initiate intracellular Janus kinase/signal transducer and activator of transcription (JAK/STAT) signal pathways to induce the expression of numerous IFN-stimulated genes (ISGs) to clear the virus [8]. Furthermore, current studies indicate that IFN could inhibit HBV replication at different steps of the viral life cycle in various ways [57]. Specifically, Robek et al. showed that the inhibitors of the proteasome can block the antiviral effect mediated by IFN [63]. Further study demonstrated that TRIM5, TRIM6, TRIM14, TRIM22, TRIM25, TRIM26, and TRIM31, which have the activity of E3 ligase that could inhibit HBV transcription, are ISGs [52, 53, 64, 65]. In addition, Tan et al. showed that TRIM14 could limit HBV replication via binding the C-terminal of HBx and blocking HBx-mediated degradation of Smc5/6 that is dependent on the HBx-DDB1-CUL4 E3 ligase complex [64]. Taken together, these reported results indicate that IFN-induced anti-HBV immune response is related to the component of the UPS.

Although IFN could eradicate the HBV, the antiviral function of IFN could be compromised by the virus. The exact mechanism for the inhibition of IFN mediated by HBV remains not well clear. However, it is estimated that interfering with the function of ISGs that are associated with the UPS contributes to the anti-IFN function of the virus. For example, HBV could repress the expression of TRIM25 to facilitate viral infection [66]. ISG15/USP18 is also an ISG, and it could conjugate to Ub, incorporate into Ub chains, and negatively regulate the turnover of ubiquitylated proteins [67]. In addition, the reports of Kim and Li et al. showed that ISG15/USP18 could promote HBV replication [68–70].

### The role of the UPS in the biological function of hepatocytes mediated by HBV

The components of the UPS and cellular proteins regulated by the UPS are involved in several cellular processes, including the cell cycle, proliferation, invasion, and apoptosis [15, 16, 71]. Therefore, the abnormal expression of cellular proteins mediated by the HBV through disrupting the function of the UPS is responsible for the abnormal biological function of HBV-related hepatocytes or hepatoma cells. For example, ISG15/USP18 promotes the proliferation and inhibits the apoptosis of HBV-associated HCC cells [72]. In addition, the HBV stimulates the gene expression of the E3 ligase Parkin and disrupts mitochondrial dynamics toward fission and mitophagy to decrease the virus-induced apoptosis in hepatocytes [73]. Liu et al. showed that HBe and its precursors could interact with NUMB, which could enter in a complex with p53 and the E3 ligase HDM2, and impair the stability of p53 mediated by Ub-dependent proteasomal degradation, to promote proliferation and inhibit apoptosis of HCC cells [74]. In addition, Hsieh et al. demonstrated that the pre-S2 LHBS mutant induces the Ub-dependent proteasomal degradation of cyclin-dependent kinase inhibitor p27 (Kip1) through interacting with the Jun activation domain-binding protein 1 (JAB1) to promote the proliferation of HCC cells [75].

**Fig. 2:**
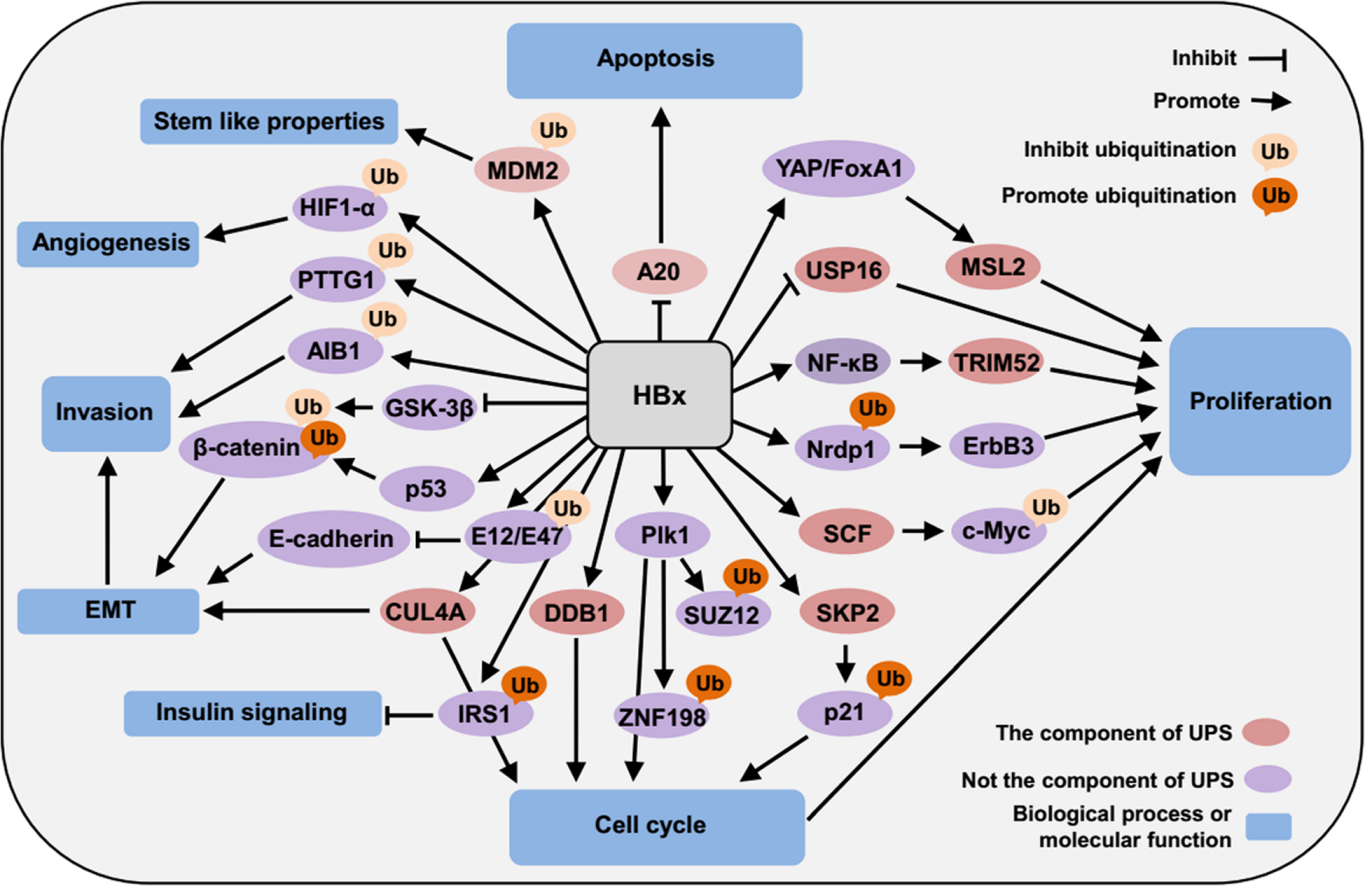
The mechanisms related to the UPS in the pathogenesis mediated by HBx. HBx could promote the development of HCC through promoting or inhibiting the functions of different components of the UPS and the ubiquitination of proteins mediated by the UPS to regulate several biological processes and molecular functions, including proliferation, apoptosis, angiogenesis, EMT, invasion, cell cycle, stem-like properties, and insulin signaling. EMT: epithelial-mesenchymal transition

HBx is considered as a multifunctional regulator with a vital role in the development of HCC [12]. Several studies have demonstrated that transgenic mice with a high HBx level could develop HCC [76]. Additionally, although HBx is detected at low levels in the liver tissue of CHB patients, it is detected at high frequency in HBV-associated HCC patients [77]. These results indicate that high-level hepatic expression of HBx is a key for the development of HBV-related HCC. Based on HBx over-expression assays in HCC cells, the protein was demonstrated to regulate the expression of cellular proteins that are mediated by the UPS to modulate a variety of biological processes (Fig. 2). For example, HBx increases TRIM52 expression via the NF-κB signal pathway [78], and elevates MSL2 based on YAP/FoxA1 signaling [49], to promote cellular proliferation. Upon binding with DDB1 and CUL4A [79, 80], HBx affects the cell cycle and induces genetic instability to favor HCC development. Plk1 is a molecule that participates in cell cycle progression. Zhang et al. revealed that HBx increased the expression of Plk1 in favor of mediating the ubiquitination of SUZ12 and ZNF198 to promote the progress of HCC [81]. c-Myc is an oncoprotein that contributes to the proliferation of HCC cells [82]. The stability of c-Myc is regulated by Skp/cullin/F-box (SCF) E3 ligase. Lee et al. found that HBx bound to SCF E3 ligase and inhibited the ubiquitination and proteasomal degradation of c-Myc [83, 84]. In addition, HBx reduces the stability of neuregulin receptor degradation protein 1 (Nrdp1) that is regulated by the proteasome to increase the expression of ErbB3 and further enhance the proliferation of HCC cells [85]. Current studies showed that HBx mutants also have been implicated in the development of HCC [86], and the components of the UPS are involved in the development of HCC mediated by HBx mutants. For instance, Huang et al. indicated that HBx with mutations in the core promoter region deregulated S phase kinase-associated protein 2 (SKP2), a member of the F-box family that acts as a substrate-specific adaptor in the SCF E3 ligase complex, to decrease the stability of its target protein p21 [87], and induce the changes in the cell cycle to facilitate the proliferation of HCC cells. Besides, Qian et al. showed that the downregulation of USP16 mediated by carboxyl-terminal truncated HBx mutants promotes the proliferation of HCC cells [88].

The protooncogene pituitary tumor-transforming gene 1 (PTTG1) is related to tumor invasiveness. In HBV-related HCC, HBx could induce the accumulation of the protein via disrupting the interaction of PTTG1 with the SCF E3 ligase complex to repress PTTG1 degradation [89]. In addition, HBx stabilizes amplified in breast cancer 1 (AIB1) protein through preventing it from proteasome-dependent degradation and cooperates with this molecule to enhance the invasiveness of HCC cells [90]. β-catenin is a critical molecule associated with epithelial-mesenchymal transition (EMT) [91], a process that contributes to the invasion of HCC cells. However, the mechanism of β-catenin upregulation in HBV-related HCC is unclear. Jung et al. showed that HBx affected the expression of β-catenin via Ub-dependent proteasome pathways that rely on p53 status. In the presence of p53, HBx increased the ubiquitination of β-catenin via the activation of a p53-Siah-1 proteasome pathway. In the absence of p53, HBx attenuated the ubiquitination of β-catenin protein via the inhibition of the glycogen synthase kinase-3β (GSK-3β) pathway [92]. E-cadherin is an adhesion molecule that could suppress EMT. Kim et al. showed that HBx is able to increase the expression of the transcriptional repressors E12/E47 by inhibiting Ub-dependent proteasomal degradation to repress E-cadherin protein and induce EMT to favor invasion of HCC [93]. In addition, CUL4A, an E3 ligase, also interacts with HBx and promotes EMT to enhance invasion of HCC cells [80].

Apart from the role in regulating the cell cycle, proliferation, and invasion, HBx also has been reported to sensitize hepatocytes to apoptosis induced by TNF-related apoptosis-inducing ligand (TRAIL) via inhibiting E3 Ligase A20 [94]. Stabilizing hypoxia-inducible factor-1α (HIF1-α) protein from proteasome-dependent degradation, HBx could induce angiogenesis [95]. In addition, early studies revealed that HBx enhanced the expression of MDM2 by directly binding with MDM2 and inhibiting its Ub-directed degradation to enhance the stem-like properties in HCC cells [96]. Recently, it has been demonstrated that HBx utilized the UPS to mediate the degradation of insulin receptor substrate 1 (IRS1) via ubiquitination to impair insulin signaling in hepatocytes [97].

## Conclusions

As described above, the UPS acts as a double-edged sword in HBV infection. On the one hand, the UPS serves as a host defense mechanism to eliminate viral components of HBV and blocks the viral life cycle. On the other hand, the UPS could be utilized by the HBV to maintain the level of viral proteins and contribute to viral replication. Furthermore, the HBV regulates the expression of cellular proteins that are involved in multiple biological processes to promote the development of liver diseases. Specifically, HBx is considered as a cancer cofactor and has been reported to be involved in both viral replication and hepatocarcinogenesis during HBV infection [98]. Although the precise mechanisms associated with the role of HBx in viral infection remain to be understood, the reviewed studies support that modulation of the host UPS by HBx plays an important role in regulating HBV replication and the pathogenesis of liver diseases, especially HCC. Significant progress has been made in identifying the molecules that target specific UPS components as a potential target for different disease therapies [99–101]. A further understanding of the complicated interactions between the HBV and the host UPS, particularly the HBx-UPS interaction, could provide novel insight into the mechanisms of viral pathogenesis, and help determine potential therapeutic strategies involving targeting the UPS to block HBV infection and associated liver diseases.

## Data Availability

Not applicable.

## Abbreviations

AIB1: breast cancer 1
cccDNA: covalently closed circular DNA
c-FLIP: Cellular FLICE inhibitory protein
cIAP2: Cellular inhibitor of apoptosis protein 2
DDB1: damaged DNA binding protein 1
DUBs: deubiquitinating enzymes
EMT: epithelial-mesenchymal transition
HBV: hepatitis B virus
HBx: hepatitis B virus X protein
HCC: hepatocellular carcinoma
IFN: interferon
IRF3: interferon regulatory factor 3
IRS1: Insulin receptor substrate 1
ISGs: IFN-stimulated genes
JAB1: Jun activation domain-binding protein 1
JAK/STAT: Janus kinase/signal transducer and activator of transcription.
MAVS: mitochondrial antiviral signaling
NIRF: Np95/ICBP90-like RING finger protein
NLRs: NOD like receptors
NTCP: sodium taurocholate cotransporting polypeptide
PA28γ: proteasomal activator 28 gamma
PAMPs: pathogen-associated molecular patterns
Par14: parvulin 14
pgRNA: pregenomic RNA
PLSCR1: phospholipid scramblase 1
PMSC8: proteasome subunit C8
PRRs: pattern recognition receptors
PTM: post-translational modification
PTTG1: protooncogene pituitary tumor-transforming gene 1
RLRs: RIG-I like receptors
Siah-1: seven in absentia homologue 1
SKP2: S phase kinase-associated protein 2
STING: stimulator of interferon genes
TLRs: toll-like receptors
TRAIL: TNF-related apoptosis-inducing ligand
Ub: ubiquitin
UPS: ubiquitin proteasome system
USP15: Ub-specific protease 15
